# Positive Influence of Behavior Change Communication on Knowledge, Attitudes, and Practices for Visceral Leishmaniasis/Kala-azar in India

**DOI:** 10.9745/GHSP-D-17-00087

**Published:** 2018-03-21

**Authors:** Raghavan Srinivasan, Tanwir Ahmad, Vidya Raghavan, Manisha Kaushik, Ramakant Pathak

**Affiliations:** a New Concept Information Systems, New Delhi, India.

## Abstract

**Background:**

Visceral leishmaniasis (VL) is endemic to 54 districts in 4 states of India. Poor awareness of the disease and inappropriate health-seeking behavior are major challenges to eliminating the disease. Between February 2016 and March 2017, we implemented a behavior change communication (BCC) intervention in 33 districts of Bihar, 4 districts of Jharkhand, and 3 districts of West Bengal using a mix of channels, including group and interpersonal communication, to improve knowledge, attitudes, and practices of communities, frontline health workers, and opinion leaders. We conducted an impact assessment in October 2016, after the second indoor residual spraying (IRS) round, in Bihar and Jharkhand to evaluate the effect of the BCC intervention.

**Methods:**

Villages in 10 districts of Bihar and 4 districts in Jharkhand were selected for inclusion in the assessment. Selected villages were categorized as either intervention or control based on where project activities were conducted. Households were randomly selected proportional to caste composition, and interviewers surveyed the head of the household on whether the house was sprayed during the last IRS round and on knowledge, attitudes, and practices related to VL. We interviewed 700 households in intervention villages and 350 households in control villages and conducted correlation analysis to explore the association between IRS refusal and socioeconomic variables, and tested for association between IRS refusal and exposure to BCC activities. Odds ratios (ORs) were calculated.

**Results:**

We reached an estimated 3.3 million contacts in Bihar and Jharkhand through the intervention's BCC activities. IRS refusal rates were significantly lower in intervention households than control households (mean=7.95% vs. 24.45%, respectively; OR, 0.27; 95% confidence interval [CI], 0.11 to 0.62; *P*<.001). Households in intervention villages were more aware than those in control villages that VL is spread by sand flies (68.4% vs. 7.4%, respectively; *P*<.001) and of IRS as an effective control measure (82.3% vs. 41.7%, respectively; *P*<.001). A greater percentage of households in intervention villages than control villages indicated they would encourage a patient to go to primary health centers for diagnosis and treatment of VL (77.0% vs. 39.4%, respectively) and to encourage others to accept IRS (78.6% vs. 44.6%, respectively; *P*<.001).

**Conclusion:**

Households that were exposed to community-based BCC activities largely using group and interpersonal communication had better knowledge, attitudes, and practices related to VL, including acceptance of IRS as a preventive measure, than households not exposed. BCC activities are thus an important component of VL elimination strategies.

## Abstract (precis)

After 8 months of behavior change communication activities, largely using group and interpersonal communication, refusal of indoor residual spraying to prevent visceral leishmaniasis was significantly lower among households in intervention villages (8%) than control villages (25%). Knowledge and attitudes were also better among the households in the intervention villages than control villages.

## BACKGROUND

Visceral leishmaniasis (VL), known as kala-azar (KA) in South Asia, is a vector-borne disease caused by the Leishmania parasite (*Leishmania donovani*), spread by the infected sand fly (*Phlebotomus argentipes*).^1^ Left untreated, it could be potentially fatal. In 2010, the World Health Organization (WHO) estimated that of 1.6 million new cases of leishmaniasis annually, 0.5 million were visceral while 1.1 million were cutaneous.^2^ As many as 90% of the new visceral cases are concentrated in Bangladesh, Brazil, Ethiopia, India, Nepal, and Sudan.

In India, VL is endemic to 54 districts spread over 4 states—Bihar, Jharkhand, Uttar Pradesh, and West Bengal. Poor awareness of the disease coupled with inadequate health-seeking behaviors are considered to be major challenges to achieving elimination of VL. In alignment with global efforts to curb menace of this neglected tropical disease, in 2014 the Government of India declared VL elimination to be a priority public health intervention and developed the *National Road Map for Kala-azar Elimination 2014*. In this policy guideline, communication and social mobilization for behavioral impact, along with integrated vector management through indoor residual spraying (IRS), are among the 5 priority elimination strategies to bring incidence of VL cases below 1 per 10,000 persons annually in India.^3^

Visceral leishmaniasis is endemic in 4 states of India.

In 2015, UK Aid formed a consortium named KalaCORE comprising 4 agencies—Mott MacDonald, Médecins Sans Frontières, the Drugs for Neglected Diseases Initiative, and the London School of Hygiene & Tropical Medicine—to strengthen public health efforts toward the elimination of VL in South Asia and Africa.^4^ Between February 2016 and March 2017, New Concept Information Systems, as a subcontractor to the consortium, implemented behavior change communication (BCC) activities to support the elimination of VL in 3 of the 4 endemic states in India. The year-long communication activities were focused on improving knowledge, attitudes, and practices (KAPs) related to VA and IRS among communities, frontline health workers, and opinion leaders. The purpose of this article is to describe the BCC intervention and assess the effect of the intervention after 8 months of implementation (March through October 2016) on VL-related KAPs, especially on IRS refusals at the household level. Two IRS rounds were conducted over this assessment period: March–May 2016 and July–September 2016. West Bengal was not included in the assessment since the KalaCORE consortium could not support BCC interventions in that state beyond 2016, while it continued supporting the BCC intervention in another phase in Bihar and Jharkhand. Therefore, this article focuses mostly on implementation of the BCC intervention and its assessment in the 2 states of Bihar and Jharkhand.

Behavior change communication activities were implemented in 3 of the 4 endemic states of India to support elimination of visceral leishmaniasis.

## PROGRAM DESCRIPTION

We implemented the BCC intervention in 33 high-endemic districts of Bihar, 4 districts of Jharkhand, and 3 districts of West Bengal (Figure). In Bihar and Jharkhand, each large village is subdivided into approximately 10–12 wards, which was the basic unit used for covering the village with BCC activities. We covered each village in a period of 3 days (1 day per village before each IRS round plus 1 day for follow-up activities at a later point in time). During any particular month during the intervention, the field team conducted community-level BCC activities over an average of 18 days per month per BCC facilitator and used the remaining 7 days for follow-up, documentation, and reporting.

**FIGURE:**
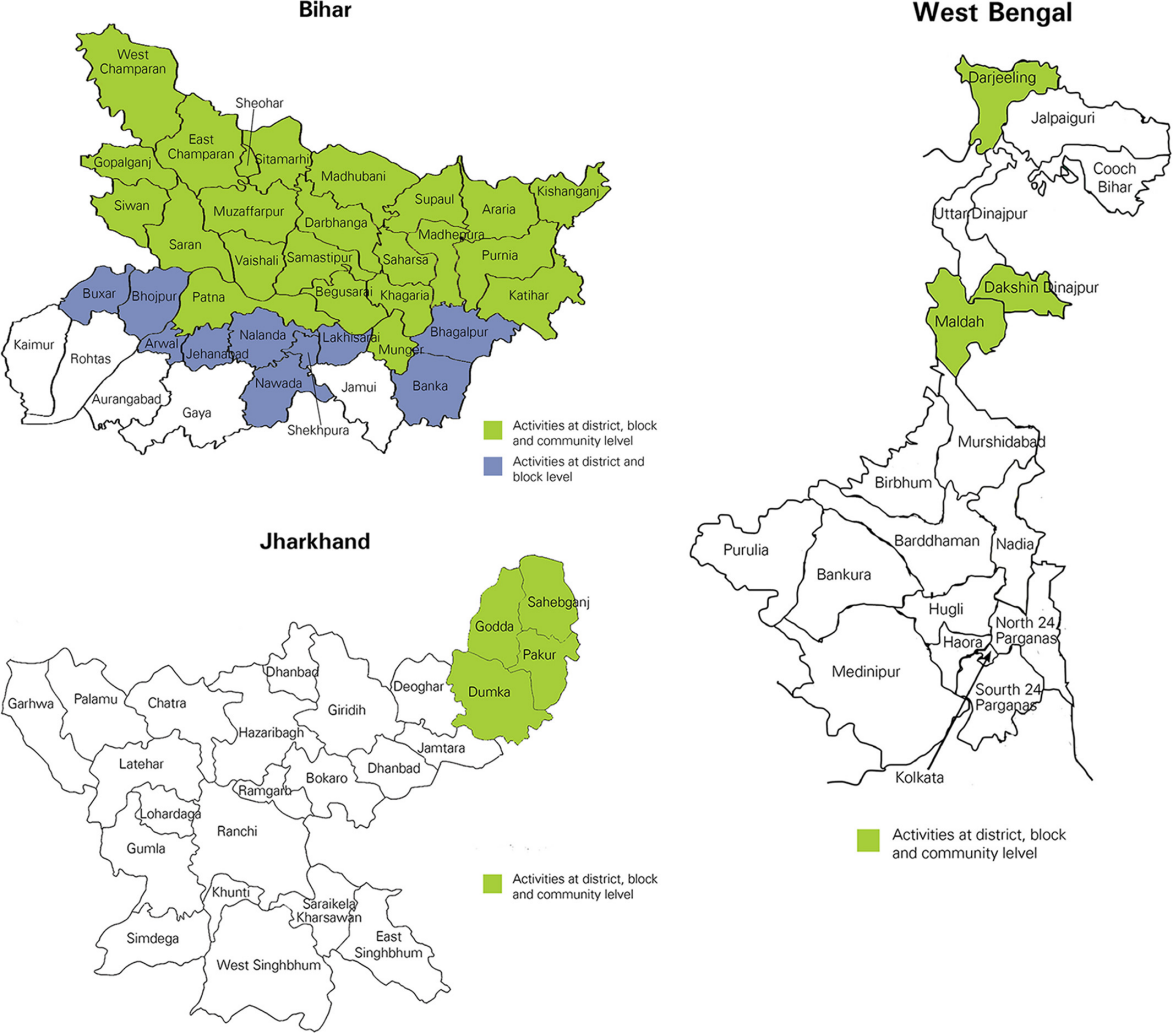
Location of Project Districts in Bihar, Jharkhand, and West Bengal States of India, February 2016 to March 2017

Aligned with the socio-ecological model of health promotion,^5^ which proposes that health is mediated by individual, interpersonal, community, organizational, social, and global forces, our communication strategy to eliminate VL was designed to address information (knowledge), motivation (attitudes, beliefs), ability to act (skill, efficacy, access), and norms (perceived, sociocultural, gender) at the individual, family, community, and institutional levels.

Key areas of focus for the BCC intervention were on improving KAPs of communities, frontline health workers, and opinion leaders about:

1. Causes, symptoms, and severity of VL
2. Diagnosis and treatment of VL and post-kala-azar dermal leishmaniasis (PKDL)—a complication of VL that is characterized by a rash in a patient who has recovered from VL
3. Prevention of VL through IRS

We mapped the current situation prevailing in the intervention districts with respect to these focal areas, gleaned from formative research conducted in Bihar, Jharkhand, and West Bengal during 2011 and 2012, to possible challenges to overcome, key BCC messages to address the challenges, and interdependencies involving capacities of frontline health workers and availability of drugs and diagnostic kits (Table 1).

**TABLE 1.** Situational Analysis According to Focal Areas of the BCC Intervention

Focal Areas	Current Situation/Issue	Barriers/Challenges	Components for BCC to Address	Interdependencies^a^
Knowledge and attitudes among communities and opinion leaders about **causes, symptoms, and severity** of VL	Unaware/not completely aware of the cause Inability to differentiate between malaria and VL in terms of causes and causative vectors	Insufficient/incorrect information about causative vector in transmission of VL	Knowledge about causes of VL and differences between malaria and VL	Building capacities of FLWs in IPC and effective use of BCC tools^b^
	Not aware of all the symptoms and the modes of transmission of VL	Insufficient/incorrect information about symptoms and modes of transmission	Knowledge about symptoms and modes of transmission	Building capacities of FLWs in IPC and effective use of BCC tools^b^
	Awareness and perception that VL is severe and can be fatal if not diagnosed and cured on time	Late diagnosis due to lack of information about symptoms Lack of identification of symptoms, leading to late diagnosis and delayed treatment	Knowledge that delayed diagnosis leads to high transmission of parasite by vector, thereby increasing the case load within a household	Building capacities of FLWs in IPC and effective use of BCC tools^b^
Knowledge, attitudes, and practices among communities and opinion leaders about **diagnosis and treatment** of VL	Analysis of health-seeking behavior of community at the onset of fever reveals that most sought home remedies or visited the local healer (*ojha*) Very few prefer going to government health facilities due to various service-delivery reasons Community is not fully aware about the Rk39 test and about where it can be done	Lack of awareness about diagnosis and treatment and about where to go Lack of timely diagnosis due to unavailability/inadequate quantity of Rk39 Lack of or poor access to government health facilities due to distance and transportation costs Low credibility of public health service providers (including FLWs) and the perception/experience of people that there are no/insufficient medicines available at these health facilities Low levels of motivation and knowledge among FLWs and other providers regarding diagnosis and treatment	Health-seeking behavior for early diagnosis and prompt treatment through public service delivery channels, emphasizing that it is of high quality and free of cost Informing the community about the various services available and how they can be accessed Increased credibility, confidence, and satisfaction among community on public health service delivery channels at the PHC and at Sadar district hospital Increased credibility, trust, and confidence in FLWs, so the community feels motivated to seek help from them	Building capacities of FLWs in IPC and effective use of BCC tools^b^ Ensuring sufficient stock of Rk39 diagnostic kits and AmBisome vials, as well as complete and appropriate treatment at Sadar district hospital Advocating with policy makers regarding implementation of guidelines on incentives for patients and FLWs for treatment Addressing ‘softer’ aspects like behavior and treatment toward patients by PHC/Sadar district hospital staff
Knowledge, attitudes, and practices among communities and opinion leaders about **prevention** of VL	Less knowledge on prevention measures of VL to prevent breeding of sand fly. Despite incomplete knowledge, VL perceived to be a preventable disease	Incomplete knowledge on the methods of prevention	Knowledge on preventive methods for Kala-azar (VL)	Building capacities of frontline functionaries in IPC skill building and effective use of BCC tools^b^
	Limited knowledge of IRS as one method of prevention Insufficient information provided to households well in advance of the date of the spray Practices related to covering the entire house through IRS, including inside the house and cowsheds and in the surroundings and outside the house	IRS has not been done in the recent past in the village Perceive the spray to affect the walls of the house and contaminate the food because of the bad smell and the stains it leaves behind Spray workers taking bribes/food grains in exchange for spraying IRS perceived to be ineffective in the long run Allergy to the smell (causes headache, cough, etc.) Face difficulty while emptying the house prior to IRS (which is related to prior communication of the IRS dates) Absence of male member in the house when spray workers arrive Delay and continuous changes in dates of IRS	Complete knowledge about IRS and its intended benefits Advantages of SP and the improvement over DDT Key influencers and opinion leaders (ward members, Mukhiya, etc.) to play an active role in demanding complete spray	Building capacities of FLWs in IPC and effective use of BCC tools^b^ Training of spray workers on technical and soft skills Ensuring dates of IRS are communicated well in advance, and adhered to by the spray squad Coordinating with other development partners like CARE
	Lack of basic awareness on maintaining cleanliness and keeping the surroundings clean as preventive methods for VL	Limited knowledge of importance/benefits of keeping household, cowsheds, and surroundings clean and dry	Knowledge and awareness of maintaining proper hygiene and cleanliness especially in damp areas	Building capacities of FLWs in IPC and effective use of BCC tools^b^
Knowledge, attitudes, and practices among communities and opinion leaders about **PKDL or relapse of kala-azar**	Inadequate awareness about PKDL and importance of treatment among patients and their families Lack of sufficient information that PKDL is a reservoir of infection, which would increase transmission and the case load Delayed reporting of PKDL cases due to lack of knowledge	Insufficient knowledge about PKDL among community members	Knowledge about PKDL and importance of getting it treated immediately	Building capacities of FLWs in IPC and effective use of BCC tools^b^ Increasing awareness and motivation about PKDL among Medical Officer In-Charge

Abbreviations: BCC, behavior change communication; FLW, frontline health worker; IPC, interpersonal communication; IRS, indoor residual spraying; PHC, primary health center; PKDL, post-kala-azar dermal leishmaniasis; VL, visceral leishmaniasis.

a Intervention focused primarily on BCC at the community level while recognizing that achieving the overall goal of VL elimination depends also on structural factors such as availability of timely and quality services.

b The intervention used BCC facilitators to implement the BCC activities but also involved FLWs in the BCC activities; no formal communication capacity building of the FLWs, however, was done.

One of the key focal areas of the BCC intervention was to improve community knowledge about prevention of visceral leishmaniasis through indoor residual spraying.

### Key Audiences

We identified primary and secondary audiences at the village, block, district, and state levels. The primary audience consisted of patients and families in endemic areas, communities living in damp and humid areas, workers in agricultural fields, and pregnant women. The secondary audience consisted of community-level opinion leaders, health workers, and policy makers (Table 2).

**TABLE 2.** Primary, Secondary, and Tertiary Audiences of BCC Activities

Level	Type of Audience	Specific Audience
Village/*tola*/community level	**Primary audience:** Will be carrying out the intended action and therefore the prime target for BCC interventions	Patients and families in the endemic areas Communities and clusters living in damp humid areas and near vegetation, especially certain vulnerable sections of the population (excluded communities and marginalized groups) Workers in agricultural fields and in cowsheds Pregnant women and families with children residing in the endemic areas
Village/*tola*/community level	**Secondary audience:** Responsible for facilitating the desired action toward successful behavior change	Community-level key influencers and opinion leaders such as PRI members, religious leaders, SHGs/AGGs/youth groups, school teachers/headmasters Children in middle and secondary schools
Village/block level	**Secondary audience:** Responsible for facilitating the desired action toward successful behavior change	MoICs, frontline health workers (if any), and active SHG women
District, state, and national level	**Tertiary audience:** Responsible for providing an enabling environment for sustained behavior change	Policy makers and program managers

Abbreviations: AGG, adolescent girls' group; BCC, behavior change communication; MoIC, Medical Officer In-Charge; PRI, Panchayati Raj Institution; SHG, self-help group.

### Communication Objectives

Table 3 maps in detail each of the audience segments to specific communication objectives (along with BCC activities and tools), which we used as a guide for training BCC facilitators. For the primary audience of community members, the communication objectives were to increase awareness about VL, appropriate health-seeking behaviors, and where to access treatment, as well as to increase awareness of IRS and of the importance of allowing the IRS team to enter the household and spray all rooms including the kitchen, prayer room, and the cow shed. For secondary audiences of frontline health workers, the communication objectives were to improve their knowledge about the importance of timely diagnosis and treatment of patients with VL, along with coverage of all households in endemic villages by the IRS spray team, and to improve their capacity to promote community awareness about VL and motivate appropriate health-seeking behaviors. For opinion leaders, including religious leaders and school teachers, the communication objectives focused on increasing their awareness of VL, mobilizing communities to access health services, and ensuring proper IRS in their villages.

**TABLE 3.** Mapping of Key Audiences to Communication Objectives, BCC Activities, and BCC Tools

Level	Audience	Communication Objectives	Description of BCC Activity	BCC Materials/Tools
Village/*tola*/community level	**Primary audience:** Patients and families in the endemic areas Communities and clusters living in damp humid areas and near vegetation Workers in agricultural fields and in cowsheds Pregnant women and families with children residing in the endemic areas	Increase awareness about VL and PKDL causes, symptoms, and mode of transmission Ensure timely identification and reporting of fever and PKDL cases to avoid delays in diagnosis and treatment (which increases chances of transmission and case load) Ensure IRS within complete household (including cowsheds, cracks, holes) Maintain cleanliness and hygiene within household and surroundings and keep them dry Increase in awareness regarding: Location and accessibility of the nearest PHC and Sadar district hospital Duration, costs, side effects regarding treatment Provision of incentives for treatment	Group communication sessions using the VL film IPC using the flip-book IPC activities such as simple and participatory games, which can be carried out without any specific BCC tool Miking during IRS (only in Bihar) Munadi (drum beating) during IRS (only in Jharkhand)	VL film Flip-book Posters and stickers displayed at the PHC and Sadar district hospital Display posters on rickshaws, tempo, and other vehicles plying in rural areas SMS alerts
Village/block level	**Secondary audience:** Frontline health workers at village level; MoIC and KTS at the block level	Ensure timely diagnosis and treatment of Kala-azar patients Ensure active case finding and identification during Kala-azar fortnights and passive case finding during home visits (both Kala-azar and PKDL) Increase community awareness on causes, symptoms, diagnosis, treatment and prevention of Kala-azar and PKDL Provide identification and motivation of patients and their families for seeking timely diagnosis and treatment for fever and PKDL (through IPC and counselling during home visits) Provide information about incentives/other entitlements for Kala-azar patients	Ensuring active participation of FLWs in group communication sessions using the Kala-azar film (to ensure continuity and sustainability) IPC using the flip-book Interactions/meetings using FAQ booklet Capacity building on IPC and communication skills	VL film for GC sessions Flip-book for IPC sessions FAQ booklet Module on IPC and effective communication SMS alerts
Village/*tola*/community level	**Secondary audience:** Opinion leaders, PRI/Gram Sabha members, religious leaders, SHGs/AGGs/youth groups, school teachers and headmasters	Increase awareness about VL and PKDL causes, symptoms, and mode of transmission Timely reporting of fever and PKDL cases Ensuring IRS of complete village in each and every household (including cowsheds) Mobilize and motivate the community to timely report PKDL cases Mobilize and motivate the community to access and demand various services Provide information and assist patients in getting incentives after treatment Provide support during active case finding in Kala-azar fortnights	Ensuring active participation in group communication sessions using the VL film IPC using the flip-book Interactions/meetings using FAQ booklet; Screenings of VL film at the school Miking during IRS (only in Bihar) *Munadi* (drum beating) during IRS (only in Jharkhand)	VL film Flip-book FAQ booklet Posters and stickers distributed to the community, the PHC, and Sadar district hospital Display posters on rickshaws, tempo, and other vehicles plying in rural areas SMS alerts
Village/block level	**Secondary audience:** Private practitioners/traditional healers	Ensure timely diagnosis and treatment of VL patients Informing the patients about causes, symptoms, diagnosis, treatment, and prevention of VL and PKDL Provide information about diagnosis and treatment processes as well as procedures for referral to Sadar district hospital Ensure proper recording and reporting of cases Inform the patients about the nearest accessible and functional health facility	Sensitization workshops	FAQ booklet Posters and stickers for display and distribution in clinics, hospitals SMS alerts Workshop kit
District, state, and national level	**Tertiary audience:** Policy makers and program managers	Provision of quality and timely resources (human, equipment, and finances) Provision of timely and regular supply of diagnostic kits and medicines Ensure proper planning and implementation to ensure complete coverage through IRS Devise a plan for capacity building of health care service providers and spray staff on technical and soft skills to enhance their motivation and awareness levels Coordinate with other departments to ensure concerted efforts toward elimination Ensure periodic review of the VL elimination program by senior officials at state and district levels	Advocacy by KalaCORE with support	Advocacy

Abbreviations: AGG, adolescent girls' groups; BCC, behavior change communication; FAQ, frequently asked questions; IPC, interpersonal communication; IRS, indoor residual spraying; KTS, Kala-azar Technical Supervisor; MoIC, Medical Officer In-Charge; PHC, primary health center; PKDL, post-kala-azar dermal leishmaniasis; PRI, Panchayati Raj Institution; SHG, self-help group; VL, visceral leishmaniasis.

### Communication Channels

The BCC intervention used a mix of media channels comprising IPC and group communication with IRS-resistant families, frontline health workers, and opinion leaders in the community; outdoor/mid-media, such as “miking” in Bihar and West Bengal (a 3-wheeler fitted with a loudspeaker travels through the villages to convey BCC messages), drum beating in Jharkhand, posters, and billboards, in villages and at the facility level to create an enabling environment; and mobile-based media. Mass media was used sparingly. The media mix was based on the results of the formative research mentioned earlier, which showed that IPC and group communication were the most effective channels to reach communities affected by this disease. Supplement 1 outlines the BCC activities, materials, and audience segments by each type of media.

The BCC intervention used a mix of media channels including interpersonal communication, group communication, mid-media, and mobile-based media.

### Communication Messages, Tools, and Activities

The messages were developed mainly around 2 broad issues: (1) prevention of VL, mainly through IRS, and (2) identifying symptoms of VL that would prompt appropriate health-seeking behavior, ultimately leading to diagnosis and treatment.

We developed several types of BCC tools, such as a film on KA, a flip-book for IPC sessions, a frequently asked questions (FAQ) booklet, posters, and stickers, for use at the village and higher levels. We pretested these tools in July 2015 with stakeholder groups in Bihar at 2 levels—the community level and with frontline health workers, as applicable. The BCC materials were piloted in 7 districts of Bihar (Araria, Vaishali, Muzaffarpur, Purnia, Sitamarhi, Saharsa and Saran) between August and November 2015. Evidence from the pilot further confirmed that most of the VL-endemic habitations were marginalized from mainstream media and typically had low literacy levels. People were largely unaware of the signs and symptoms of VL and of preventive measures. They were also unaware of the single-day treatment of choice (injectable liposomal amphotericin B [LAmB]), which for most positive cases is more beneficial than the previous standard 28-day treatment (oral miltefosine) because it drastically reduces the number of sick days and associated indirect costs (e.g., wages lost for the patient and accompanying relative, days lost, transport, food, lodging costs).^6^ Community members were also unaware of the differences between malaria and VL, government incentives for obtaining VL treatment, the role of frontline health workers in supporting VL diagnosis and treatment, and the benefits of IRS of houses with synthetic pyrethroid (SP), which does not leave odor and white patches after spraying. Two of the most critical issues identified were (1) low acceptance of IRS due to earlier negative experiences with DDT sprays such as odor, white patches left on walls and wooden household articles, and increased insect levels after the spray, and (2) lack of trust in the benefits of the spraying process. Identification of suspected cases and immediate referral for diagnosis were not being done adequately by community-level health outreach workers. Pretesting among people residing in VL-endemic villages confirmed that group communication using the short film on VL; IPC using the flip-book by BCC facilitators and frontline health workers; and IPC with key opinion leaders such as village heads, retired government employees, political representatives, social workers, and women's group leaders using leaflets were effective in addressing such barriers.

IPC and group communication tools such as the flip-book and the KA film contained all the key messages and were used by well-trained BCC facilitators. Outdoor media materials, such as posters, stickers, and billboards, focused either on prevention through IRS or on identifying symptoms to ensure timely diagnosis and treatment. BCC materials on prevention through IRS were used to a greater degree just before or during the IRS rounds, whereas materials related to symptoms, diagnosis, and treatment were used throughout the year.

BCC activities consisted mainly of:

- Group communication sessions in the community using the KA film
- Engagement of students and teachers by screening the KA film in schools
- IPC sessions with key opinion leaders in the village using the flip-book and FAQ booklet
- General awareness-raising through outdoor media (posters, stickers, billboards)
- Sensitization of frontline health workers on VL by screening the KA film and training on proper use of the flip-book

For the intervention states we developed implementation plans detailing the number of activities expected to be carried out and coverage targets to ensure we achieved our intended outreach goals during the implementation period. See Supplement 2 for an illustrative example for Bihar.

### Team Composition and Management

An implementation team of 216 professionals based in the intervention states carried out the intervention comprising 168 BCC facilitators, 27 district project managers, 18 quality monitors, and 3 state program managers. A team leader headed the implementation team, closely assisted by a project manager and project coordinator, both of whom were located in New Delhi (Supplement 3). They traveled frequently to the states for review meetings and monitoring visits. In addition, a team of senior resource persons with expertise in communication and capacity building helped to strengthen the capacity of the project team; these senior resource persons also conducted monitoring visits.

At the state level, the implementation teams were led by a state project manager; the state project manager for Bihar also served as the regional manager for all 3 states. The state project managers managed all tasks associated within their respective states. District project managers managed communication activities in the districts and coordinated with the District Vector-Borne Disease Control Officers and Vector-Borne Disease consultants. The implementation team also included district-level quality monitors who were in charge of supportive supervision of BCC facilitators and the BCC facilitators themselves, who were primarily in charge of conducting BCC activities at the community level.

The BCC facilitators received training over 5 days in planning and carrying out communication activities. The training covered technical information on VL, communication skills, principles of behavior change, and use of various BCC tools. During the first 2 days of training, trainers walked participants through various sessions using training job aids. This helped in clarifying doubts and gave ample time for activities such as question and answer sessions to gain confidence and understand how BCC activities should be conducted. The remaining 3 days were used for conducting mock sessions, where participants were asked to facilitate a community session. They were closely observed and graded based on specific parameters to help in final selection. (See Supplement 4 for field instructions.)

### Project Management Information System

An online management information system (MIS) was created to capture the project process and measure outputs (Supplement 5). BCC facilitators documented daily activities in village visit formats, which were then keyed into the online MIS on a weekly basis by district project managers. The quality of village activities was randomly checked by the quality monitors through on-the-spot monitoring and post-session assessment and reported through 40 quality-related questions. This was done using Android-based smartphones. Monitoring data were integrated into the online reporting system at the central level.

## METHODS

After the second IRS round (July to September 2016), we conducted a cross-sectional study in October 2016 in 14 VL-endemic districts (10 districts of Bihar and 4 districts of Jharkhand). The study did not include West Bengal, the third intervention state, since the KalaCORE consortium did not have funds to support activities in the state beyond 2016. The study aimed to quantify the outcomes of the BCC activities related to IRS refusal and community-level indicators related to the causative agent, preventive measures, suspected case detection, and diagnosis and treatment-seeking behavior.

The sampling method was driven by the need to include a representative set of households for analysis. First, we selected 1 high-endemic block in each of the 14 study districts based on 2 criteria: (1) the villages in the selected block had higher reported VL cases than villages in other blocks, and (2) the villages in the selected block that had IRS conducted. We obtained the list of VL-endemic villages from the second IRS round microplan, prepared by the District Vector-Borne Disease Control Offices. From this list, we extracted those villages that were covered through the project's BCC activities. This list formed the sampling frame for selection of intervention villages to be included in the assessment, from which we randomly selected 10 villages per study district for inclusion in the assessment. Five control villages per study district were selected from the remaining villages that were not considered for the intervention sample (i.e., from the villages that were not covered by the project's BCC activities). We ensured that control villages were sufficiently distant from intervention villages in order to exclude, or at least minimize, the effect of diffusion of BCC activities into control villages. Since both intervention and control villages were selected from the list of VL-endemic villages, we considered it safe to assume that the populations had similar demographic and socioeconomic characteristics.

After categorizing the selected villages as intervention or control, households in the intervention and control villages were selected proportional to caste composition. Caste is an age-old social stratification in India, which categorizes households into upper (general) and lower caste. The lower castes consist of Other Backward Caste, Scheduled Caste, and Scheduled Tribe.^7^ In Bihar, we surveyed 3 Scheduled Caste households, 1 Other Backward Caste household, and 1 general caste household in each village. In Jharkhand, a state with a sizable tribal population, we surveyed 2 Scheduled Tribe households, 1 Scheduled Caste household, 1 Other Backward Caste household, and 1 general caste household in each village. We also ensured inclusion of households below the poverty level as well as those above the poverty level. The criteria for classification of households based on poverty level has been defined by the C. Rangarajan Committee, Government of India (2014). Households with persons earning less than Rs 32 (less than US$0.50) per day in rural areas are considered below the poverty level.^8^ To account for religious diversity, we also made an effort to include households belonging to Christian, Muslim, Hindu, and Sarna faiths, and houses located at a distance from each other were selected in order to capture any diversity within the settlement.

In each study district, we surveyed 5 households each in 10 intervention villages (N=700 intervention households in all 14 study districts) and 5 households each in 5 control villages (N=350 control households in all 14 study districts). We based this sample size on a power calculation using IRS refusal rates that were available from government sources from the previous round of IRS. Only half as many control households were sampled as intervention households due to limited time and resources.

The survey, administered with the house owner/head of the family, included questions on socioeconomic profile, IRS coverage, exposure to BCC activities, and KAPs. Those houses with rooms partially sprayed, not sprayed at all, or locked and not visited by the spray team were grouped under the category of “houses with IRS refusal” for this study.

We used the paired *t* test to test for statistically significant differences between IRS refusal among households in villages with BCC activities (N=700 households) and households in villages without BCC activities (N=350 households). Odds ratios (ORs) were calculated with respect to the rate of IRS refusal for households exposed to BCC interventions in comparison with those not exposed. The assessment of KAPs was based on percentage responses.

We used the statistical package, Stata Version 11.2 (Stata Corp LP, Texas, USA) for the quantitative analysis, and Microsoft Excel 2011 for qualitative analysis. To calculate ORs, we used the trial version of MedCalc (Ostend, Belgium), an online easy-to-use statistical software.^9^

### Ethical Considerations

As the study was not biomedical in nature, involved less than minimal risk, and was meant solely for academic purposes, formal Institutional Review Board approval was not required per ethical guidelines of the Indian Council of Medical Research (ICMR). Further, as the study was related to a public health program with community (consumer) acceptance, it qualified for waiver from the formal consent process from the KalaCORE consortium. Verbal informed consent was obtained prior to interviews and unique code numbers were assigned to each respondent to maintain their confidentiality.

## RESULTS

### Reach of BCC Activities

Among all 3 intervention states, we estimate that we reached around 65% of the 12,265 VL-endemic villages, comprising a population of 10 million, with communication messages about VL. Based on the number of BCC activities conducted and documented in our project MIS, we estimate reaching 3.3 million contacts in Bihar and Jharkhand alone (Table 4).

**TABLE 4.** Estimated Reach of BCC Activities in Bihar and Jharkhand, India, February 2016 to March 2017

BCC Activities	No. of Activities	No. of Contacts Made^a^
Group communication sessions	24,572	982,880
VL film screenings	3,090	185,400
Interaction with frontline health workers through FAQ booklet and with KI using leaflet	64,484	64,484
IPC sessions through flip-book	74,452	595,616
Posters (on treatment, IRS, PKDL)	91,228	456,140
Wall stickers (on treatment and PKDL)	215,697	1,078,485
**TOTAL**		**3,363,005**

Abbreviations: BCC, behavior change communication; FAQ, frequently asked questions; IPC, interpersonal communication; IRS, indoor residual spraying; PKDL, post-kala-azar dermal leishmaniasis; VL, visceral leishmaniasis.

a These do not necessarily represent unique contacts because there may have been overlap in the people exposed to different BCC activities.

### Background Characteristics of Surveyed Households

The average distance between intervention households and the nearest PHC was 10.1 km, and for control households 11.9 km (Table 5). The average family size per intervention household was 8.4 and 8.9 for control households. Agriculture was the mainstay occupation followed by wage labor. In the state of Bihar, most of the households in the intervention and control households were Hindu and largely from the Other Backward Caste and Scheduled Caste. In contrast, in Jharkhand the majority of the households were split between Hindu, Christian, and Sarna (indigenous group of religions) faiths and the large majority belonged to the Scheduled Tribe.

**TABLE 5.** Demographic Characteristics of Households Included in the Survey, Bihar and Jharkhand States of India, 2016

Variables	Bihar		Jharkhand		Total	
	Control (n=250)	Intervention (n=500)	Control (n=100)	Intervention (n=200)	Control (N=350)	Intervention (N=700)
Population of villages^a^	112,522	394,497	12,590	37,516	125,112	432,013
Total no. of households^a^	24,431	63,944	2471	7620	26,902	71,564
Distance to nearest PHC, mean (km)	11.0	9.0	14.3	12.6	11.9	10.1
Average no. of family members in the surveyed households	10.1	9.5	6.0	5.6	8.9	8.4
**Caste group, %**						
General	12.8	13.4	0.0	0.5	9.1	9.7
Other Backward Caste	42.0	43.6	10.0	13.0	32.9	34.9
Scheduled Caste	31.2	27.2	9.0	3.5	24.9	20.4
Scheduled Tribe	6.4	8.2	78.0	82.0	26.9	29.3
Mahadalit^b^	6.8	5.8	1.0	0.0	5.1	4.1
Not disclosed	0.8	1.8	2.0	1.0	1.1	1.6
**Religious affiliation, %**						
Hindu	85.2	83.8	37.0	41.5	71.4	71.7
Muslim	13.6	15.0	0.0	0.5	9.7	10.9
Christian	0.8	0.4	35.0	33.5	10.6	9.9
Sikh	0.0	0.0	0.0	0.0	0.0	0.0
Jain	0.0	0.0	0.0	0.0	0.0	0.0
Buddhist	0.0	0.0	1.0	0.0	0.3	0.0
Sarna	0.0	0.6	27.0	23.5	7.7	7.1
Not disclosed	0.4	0.2	0.0	1.0	0.3	0.4
**Major occupation, %**						
Agriculture	32.4	31.3	95.0	87.5	50.3	47.4
Labor	44.8	44.1	1.0	2.5	32.3	32.2
Service	5.6	5.2	2.0	2.0	4.6	4.3
Business	10.0	11.2	1.0	2.0	7.4	8.6
Other	7.2	8.2	1.0	6.0	5.4	7.6
**Income category, %**						
Below the poverty level	74.0	70.6	90.0	86.5	78.6	75.1
Above the poverty level	22.0	25.4	9.0	12.5	18.3	21.7
Don't know	4.0	4.0	1.0	1.0	3.1	3.1

Abbreviation: PHC, primary health center.

a Data from government IRS microplan.

b Lowest Scheduled Caste subcategory.

### Exposure to BCC Activities

Nearly 69% of households in intervention villages recalled communication activities related to VL compared with only 21% in control villages (Table 6). The most commonly recalled source of communication among households in intervention villages was BCC project activities in general (24.5%), followed by posters (10.5%), miking or drum beating (6.5%), TV (6.4%), and door-to-door meetings (5.7%). (Note that only the first source mentioned by the respondent was recorded.) Respondents were also asked if they had received prior information about IRS, referring to miking conducted on the day of or before the spray to announce the arrival of the spray team. The government usually conducts these miking activities in all villages, but the BCC project team assumed responsibility for this activity in the intervention villages during the project period. A higher percentage of households in intervention villages than control villages responded positively to this question (66.9% vs. 30.3%; *P*<.001).

**TABLE 6.** Exposure to the VL Messages Among Intervention and Control Households, Bihar and Jharkhand States of India, 2016

	Intervention (%) (N=700)	Control (%) (N=350)	OR	95% CI	*P* Value
**Have heard/seen anything about VL in the last 12 months?**	68.7	21.1	8.4	(4.41, 15.90)	<.001
**Where did you hear/see anything about VL?** ^a^					
Radio	0.3	0.7	0.3	(0.01, 8.20)	.50
TV	6.4	1.3	6.3	(0.75, 53.48)	.09
Newspaper	0.5	0.7	1.0	(0.06, 16.21)	1.00
Poster	10.5	0.9	12.2	(1.55, 96.68)	.02
Health meeting at PHC	0.2	0.4	1.0	(0.02, 50.89)	1.00
Community meeting	2.9	0.4	7.2	(0.37, 141.53)	.19
Religious place/religious leaders	0.3	0.0	1.0	(0.02, 50.89)	1.00
Community leaders	0.0	0.1	1.0	(0.02, 50.89)	1.00
Friends/neighbor	1.9	1.4	2.0	(0.18, 22.65)	.57
Miking/drum beating	6.5	4.4	1.5	(0.42, 5.60)	.52
ASHA, ANM, AWW, or other health staff	3.5	2.1	1.5	(0.25, 9.27)	.65
Door-to-door meeting	5.7	0.0	13.8	(0.77, 248.81)	.07
Other	0.0	0.4	1.0	(0.02, 50.89)	1.00
BCC project activities	24.5	0.3	67.9	(4.02, 113.00)	<.001
Don't know/not heard or seen	36.8	87.0	0.1	(0.04, 0.18)	<.001
**Did you get prior information about IRS of your house?** ^b^					
Yes	66.9	30.3	4.7	(2.61, 8.61)	<.001
No	25.3	51.4	0.3	(0.18, 0.58)	<.001
Don't know	7.9	18.3	0.4	(0.16, 0.96)	.04

Abbreviations: ANM, auxillary nurse-midwife; ASHA, Accredited Social Health Activist; AWW, Agaanwadi Worker; BCC, behavior change communication; CI, confidence interval; OR, odds ratio; PHC, primary health center; PKDL, post-Kala-azar dermal leishmaniasis; VL, visceral leishmaniasis.

a Respondents were asked open-ended questions and their first response was recorded.

b Refers to information through miking on the day of or before the IRS spray to announce arrival of the spray team. In intervention villages, miking was conducted by the BCC project, whereas in control villages it was conducted by the government.

Nearly 69% of households in intervention villages recalled communication activities related to visceral leishmaniasis compared with only 21% in control villages.

### IRS Refusals

There were marked differences in the IRS refusal rate, during the second round of IRS, between intervention and control households (Table 7). Households in intervention villages exhibited a significantly lower IRS refusal rate (7.95%) compared with households in control villages (24.45%). The odds of IRS refusal were 27% less in intervention households than control households (OR=0.27; 95% CI: 0.11, 0.62; *P*<.001).

**TABLE 7.** IRS Refusal Rates During the Second Spray Round Among Intervention and Control Households, by District and Block, Bihar and Jharkhand States of India, 2016

District	Block	% IRS Refusal		OR^a^	95% CI	*P* Value
		Intervention	Control			
**Bihar**		6.20	20.90	0.24	(0.09, 0.62)	<.001
Araria	Forbesganj	5.63	51.39	0.06	(0.02, 0.15)	<.001
Gopalganj	Baruali	3.16	15.08	0.18	(0.05, 0.63)	.01
Katihar	Kadwa	3.68	4.62	0.79	(0.21, 3.04)	.73
Muzaffarpur	Paroo	11.96	16.65	0.67	(0.30, 1.48)	.32
Purnia	Kaswa	5.18	1.67	2.58	(0.49, 13.62)	.26
Samastipur	Sarairanjan	12.72	26.11	0.43	(0.20, 0.89)	.02
Saran	Dariyapur, Garkha	9.08	32.84	0.20	(0.09, 0.45)	<.001
Sitamarhi	Dumra	5.63	37.79	0.10	(0.04, 0.26)	<.001
Siwan	Barhariya	3.96	12.44	0.31	(0.10, 0.98)	.05
Vaishali	Mahua	1.44	10.12	0.09	(0.01, 0.72)	.02
**Jharkhand**		12.20	33.40	0.28	(0.13, 0.58)	<.001
Dumka	Ramgarh	1.18	34.72	0.02	(0.00, 0.14)	<.001
Godda	Sundarpahari	18.76	44.73	0.29	(0.15, 0.54)	<.001
Pakur	Littipara	19.07	25.07	0.70	(0.36, 1.38)	.31
Sahibganj	Borio	9.82	29.09	0.27	(0.12, 0.60)	<.001
**Total (Bihar and Jharkhand)**		**7.95**	**24.45**	**0.27**	**(0.11, 0.62)**	**<.001**

Abbreviations: CI, confidence interval; OR, odds ratio; IRS, indoor residual spraying.

a OR estimated based on assumption that the percentage of households that accepted IRS in the intervention areas would have refused IRS had they not been exposed to the BCC intervention. For example, in Araria district, 5.63% of households exposed to BCC activities still refused IRS. Therefore, we assume that 94.37% of households would have refused IRS if they had not been exposed to the BCC intervention, keeping aside confounders and outliers.

IRS refusal among households in intervention villages was about 8% compared with about 25% among households in control villages.

IRS refusal rates in intervention households ranged in the districts from 1% to 19%. Among control households, the rate of IRS refusal ranged from 2% to 51%—substantially higher than in intervention households (Table 7).

### Knowledge, Attitudes, and Practices

The difference in KAPs related to prevention and treatment of VL between households in intervention and control villages was pronounced (Table 8). Households in BCC intervention villages were better informed and had greater knowledge of VL compared with households in non-BCC villages, particularly in terms of their knowledge of the causes and symptoms of VL and the single-day treatment preference. For example, 68.4% of households in intervention villages knew that VL was spread by sand flies compared with only 7.4% of households in control villages (*P*<.001). Similarly, 64.7% of households in intervention villages indicated effective treatment for VL is a 1-day course of medicine provided at a government hospital compared with only 13.1% of households in control villages (*P*<.001). Furthermore, 82.3% of households in intervention villages knew that IRS was an effective preventive measure against VL compared with 41.7% of households in control villages (*P*<.001).

**TABLE 8.** Knowledge, Attitudes, and Practices^a^ Related to Prevention of VL Among Intervention and Control Households, Bihar and Jharkhand States of India, 2016

	Intervention(%) (N=700)	Control(%) (N=350)	OR	95% CI	*P* Value
**KNOWLEDGE** ^b^					
**What causes VL?**					
Insects	3.6	4.9	0.8	(0.21, 3.04)	.73
Mosquitos	20.3	63.1	0.1	(0.08, 0.28)	<.001
*Sand fly*	68.4	7.4	28.2	(11.76, 67.77)	<.001
Other	3.3	1.7	1.5	(0.25, 9.27)	.65
Don't know	4.3	22.9	0.1	(0.05, 0.42)	<.001
**Is VL contagious and spread by touching?**					
Yes	21.0	23.4	0.9	(0.46, 1.74)	.73
*No*	66.7	44.6	2.5	(1.41, 4.40)	<.001
Don't know	12.3	32.0	0.3	(0.14, 0.60)	<.001
**What are the symptoms of VL?**					
*Fever >2 weeks*	25.4	10.5	3.0	(1.36, 6.64)	.01
Loss of appetite	15.5	7.1	2.5	(0.99, 6.45)	.05
Enlargement of spleen	14.8	5.5	2.8	(1.03, 7.45)	.04
Weakness and anemia	11.4	4.2	3.0	(0.91, 9.66)	.07
Don't know	29.4	68.1	0.2	(0.11, 0.35)	<.001
**Do you know IRS prevents VL?**					
*Yes*	82.3	41.7	6.3	(3.29, 12.01)	<.001
No	7.4	19.7	0.3	(0.12, 0.75)	.01
Don't know	10.3	38.3	0.2	(0.08, 0.39)	<.001
**What is effective treatment of VL?**					
Local/traditional treatment	6.4	12.6	0.4	(0.15, 1.17)	.10
Malarial medicine	8.1	14.6	0.5	(0.19, 1.22)	.13
*1-day medicine that is given in government hospital*	64.7	13.1	12.4	(6.09, 25.36)	<.001
No need for medicine	0.6	0.0	3.0	(0.12, 75.28)	.50
Other	1.6	7.7	0.2	(0.05, 1.13)	.07
Don't know	18.4	52.0	0.2	(0.11, 0.39)	<.001
**Do you know that complete treatment of VL is available?**					
*Yes*	88.3	62.0	4.5	(2.17, 9.29)	<.001
No	3.9	14.0	0.3	(0.08, 0.81)	.02
Don't know	7.9	24.0	0.3	(0.12, 0.65)	<.001
**Do you know that complete treatment of VL is free?**					
*Yes*	81.0	39.1	6.7	(3.51, 12.66)	<.001
No	6.9	26.9	0.2	(0.08, 0.49)	<.001
Don't know	12.0	34.0	0.3	(0.13, 0.55)	<.001
**When to treat a patient with VL?**					
*Immediately*	38.0	20.3	2.5	(1.31, 4.63)	.01
Within 1 week	11.3	5.7	1.9	(0.68, 5.46)	.21
Within 2 weeks	22.6	6.0	4.7	(1.81, 12.07)	<.001
When the patient has a fever	10.4	12.6	0.7	(0.31, 1.78)	.51
Other	1.7	3.4	0.7	(0.11, 4.04)	.65
Don't know	16.0	51.4	0.2	(0.09, 0.36)	<.001
**ATTITUDES**					
**To whom do you advise patients with VL symptoms to go for diagnosis and treatment?**					
PHC	77.0	39.4	5.2	(2.83, 9.69)	<.001
Private doctor	7.3	27.1	0.2	(0.08, 0.49)	<.001
RMP/Quack	1.1	6.3	0.1	(0.01, 1.04)	.05
Traditional healer	0.4	0.3	1.0	(0.02, 50.89)	1.00
Other	1.3	2.3	0.5	(0.04, 5.55)	.57
Don't know	12.9	24.6	0.4	(0.21, 0.94)	.03
**Will you motivate/help community members to accept IRS?**					
Yes	78.6	44.6	4.6	(2.47, 8.56)	<.001
No	14.9	31.1	0.4	(0.19, 0.79)	.01
Don't know	6.6	24.3	0.2	(0.09, 0.58)	<.001
**Will you help community members to identify suspected cases of VL?**					
Yes	72.3	30.9	5.7	(3.11, 10.52)	<.001
No	18.4	44.3	0.3	(0.15, 0.53)	<.001
Don't know	9.1	24.9	0.3	(0.13, 0.67)	<.001
**PRACTICES**					
**Did you allow spraying of SP last time**^c^ **in your house?**					
Yes, all rooms	77.3	54.6	2.7	(1.49, 5.04)	<.001
Yes, partially	16.3	27.4	0.5	(0.26, 1.03)	.06
No	1.1	4.0	0.2	(0.03, 2.21)	.21
My house was locked	1.9	2.9	0.7	(0.11, 4.04)	.65
Unaware about day of IRS	3.4	11.1	0.3	(0.06, 0.93)	.04

Abbreviations: ANM, auxillary nurse-midwife; ASHA, Accredited Social Health Activist; AWW, Anganwadi Worker; CI, confidence interval; OR, odds ratio; PHC, primary health center; PKDL, post-Kala-azar dermal leishmaniasis; RMP, registered medical practitioner; SP, synthetic pyrethroid; VL, visceral leishmaniasis.

a Respondents were asked open-ended questions and their first response was recorded.

b Correct answers are shown in italics.

c Refers to the first IRS round.

Households in BCC intervention villages were better informed about visceral leishmaniasis compared with households in control villages.

When asked where they would guide a patient to go for diagnosis and treatment of VL, households in intervention villages preferred PHCs over private doctors or traditional healers (77.0% vs. 7.3% and 0.4%, respectively). In contrast, households in control villages were more evenly split between PHCs and private doctors (39.4% and 27.1%, respectively). A higher percentage of households in intervention than control villages indicated they would encourage others to accept IRS (78.6% vs. 44.6%, respectively; *P*<.001), and to help community members identify suspected cases of VL (72.3% vs. 30.9%, respectively; *P*<.001). Finally, 77.3% of households in intervention villages said they allowed spraying of all rooms during the first round of IRS compared with 54.6% of households in control villages (*P*<.001).

## DISCUSSION

Community participation in controlling and eliminating VL in endemic districts of India is crucial.^10^ Communication and social mobilization for behavioral impact and integrated vector management are among the 5 elimination strategies prioritized by the Government of India in its *National Road Map for Kala-azar Elimination 2014* and accordingly adopted in state-level public health initiatives to contain this neglected vector-borne disease. Our evaluation found that households in VL-endemic villages exposed to health communication activities had greater acceptance of IRS, awareness of the disease, and willingness to prevent and treat it compared with households in VL-endemic villages that were not targeted with these communication activities. Since IPC and group communication channels were used to reach primary stakeholder groups directly and both contained holistic information, it is difficult to attribute the contribution of specific channels to improvement in BCC indicators. A similar study in Mexico found that community understanding about the objectives of spraying were correlated with acceptance, thus leading to higher spray coverage.^11^

Households in villages exposed to BCC activities had greater acceptance of IRS, awareness of the disease, and willingness to prevent and treat it compared with households in villages not targeted with the BCC activities.

Our evaluation suggests that a short spurt of communication activities over 8 months can bring about significant positive changes in knowledge, attitudes, and practices essential for VL elimination. The BCC intervention, however, was highly resource-intensive with hundreds of BCC facilitators covering thousands of villages before, during, and after the spray months. Because this level of effort cannot be sustained over the long-term, this intensive intervention needs to be followed up with a more focused intervention that prioritizes highly endemic pockets/villages so that households do not relapse to pre-intervention levels. Government planning should factor in such intensive spurts of BCC activities followed by focused interventions and continuous outreach activities.

In addition, the government can consider strengthening linkages between outreach workers and doctors and technicians at public health centers and hospitals to better improve diagnosis and treatment of suspected VL cases. The BCC intervention seems to have resulted in an immediate increase in detection of potential VL cases, but the project did not track suspected cases to ensure diagnosis and complete treatment. Therefore, it was not possible to determine whether BCC activities ultimately led to complete treatment. Longer-term planning and implementation could create the necessary linkages between communication activities and diagnosis and treatment services to better track the prevention and care process.

### Limitations

Considering that more than 65% of VL-endemic villages of project states were covered in the year-long BCC intervention, the sample size of households from intervention and control villages included in the evaluation was relatively small. We decided to sample only half as many households in the control villages as the intervention villages, since we had limited time and resources to complete the survey. In Jharkhand particularly, households were spread out making it more difficult to implement the survey efficiently. Furthermore, sampling was not conducted in a strictly random manner. Instead, effort was made to include a representative sample of households in terms of caste, religion, occupation, and income levels. Therefore, we used a stratified sampling approach whereby sub-lists of eligible households were made at each level and then randomly selected the sample from these sub-lists. Finally, efforts were taken to make sure that control villages were sufficiently far away from intervention villages to eliminate or minimize effects of contamination. However, since both intervention and control households could have used the same public health center, contamination cannot be totally ruled out. Even with all of these sampling constraints, the sizeable difference in the IRS refusal rate between intervention and control households (7.95% vs. 24.45%, respectively) suggests that the BCC intervention had some impact on the key outcome of interest.

Another challenge of the study was to ensure completion of household surveys within 2 months after the second IRS round so that IRS recall among households was not poor. This was overcome through a combination of orientation training, administration of close-ended questionnaires, and rigorous monitoring during survey administration.

Finally, the evaluation was conducted with the inherent assumption that IRS was well-planned and executed and that the spray team was highly motivated and adopted proper spraying logistics with regular supervision by spray supervisors and quality external monitoring. However, such ideal implementation conditions may not always hold true in all VL-endemic villages, which affects the generalizability of our findings. In other words, if implementation of the IRS is poorly conducted, then communication activities may not make a difference, regardless of whether the communication activities are conducted well. Thus, further enquiry into IRS planning, the implementation process, and coverage quantification may throw more light on the actual coverage vis-à-vis houses that refused or allowed partial spraying.^12^

## CONCLUSION

Households exposed to BCC activities had significantly better awareness and acceptance of IRS than households not exposed to BCC activities, and they were better able to identify suspected VL cases and to immediately seek diagnosis and treatment at PHCs. Thus, health communication that encourages community participation should continue to be an important component of India's VL elimination strategy.^13^ To ensure sustained behavior change, BCC interventions should be planned with a longer time frame than the 12-month intervention period described here since social and behavior change is a complex process, involving several steps to transition from awareness to practice.

## Supplementary Material

17-00087-Srinivasan-Supplement5.pdf

17-00087-Srinivasan-Supplement3.pdf

17-00087-Srinivasan-Supplement2.pdf

17-00087-Srinivasan-Supplement1.pdf

17-00087-Srinivasan-Supplement4.pdf

## Notes

**Peer Reviewed**

**Funding:** UKAid through Kalacore consortium.

**First Published Online:** January 31, 2018
