# Supplementary material for: Positive Influence of Behavior Change Communication on Knowledge, Attitudes, and Practices for Visceral Leishmaniasis/Kala-azar in India
Source: Glob Health Sci Pract. 2018 Mar 21;6(1):192–209. doi: 10.9745/GHSP-D-17-00087 (PMC5878072; doi:10.9745/GHSP-D-17-00087)
Supplement: 17-00087-Srinivasan-Supplement5.pdf [file 17-00087-Srinivasan-Supplement5.pdf]

Srinivasan R, Ahmad T, Raghavan V, Kaushik M, Pathak R. Positive influence of behavior change communication on knowledge, attitudes, and practices for visceral leishmaniasis/Kala-azar in India. *Glob Health Sci Pract*. 2018;6(1). https://doi.org/10.9745/GHSP-D-17-00087

SUPPLEMENT 5. Project Management Information System

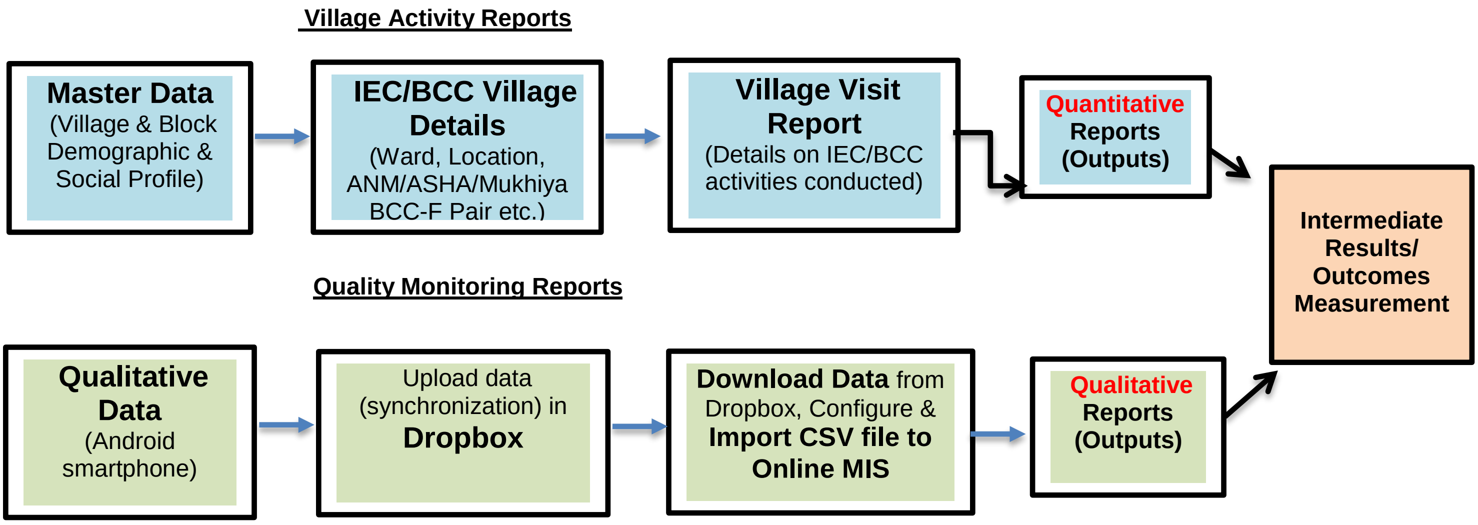

Abbreviations: ANM, auxiliary nurse-midwife; ASHA, accredited social health activist; BCC, behavior change communication; BCC-F, behavior change communication-facilitator; CSV, comma-separated values; IEC, information, education, and communication; MIS, management information system.
