# Supplementary figures and images for: Positive Influence of Behavior Change Communication on Knowledge, Attitudes, and Practices for Visceral Leishmaniasis/Kala-azar in India
Source: Glob Health Sci Pract. 2018 Mar 21;6(1):192–209. doi: 10.9745/GHSP-D-17-00087 (PMC5878072; doi:10.9745/GHSP-D-17-00087)

**SUPPLEMENT 3.** Organogram of the Bihar and Jharkhand Project Teams

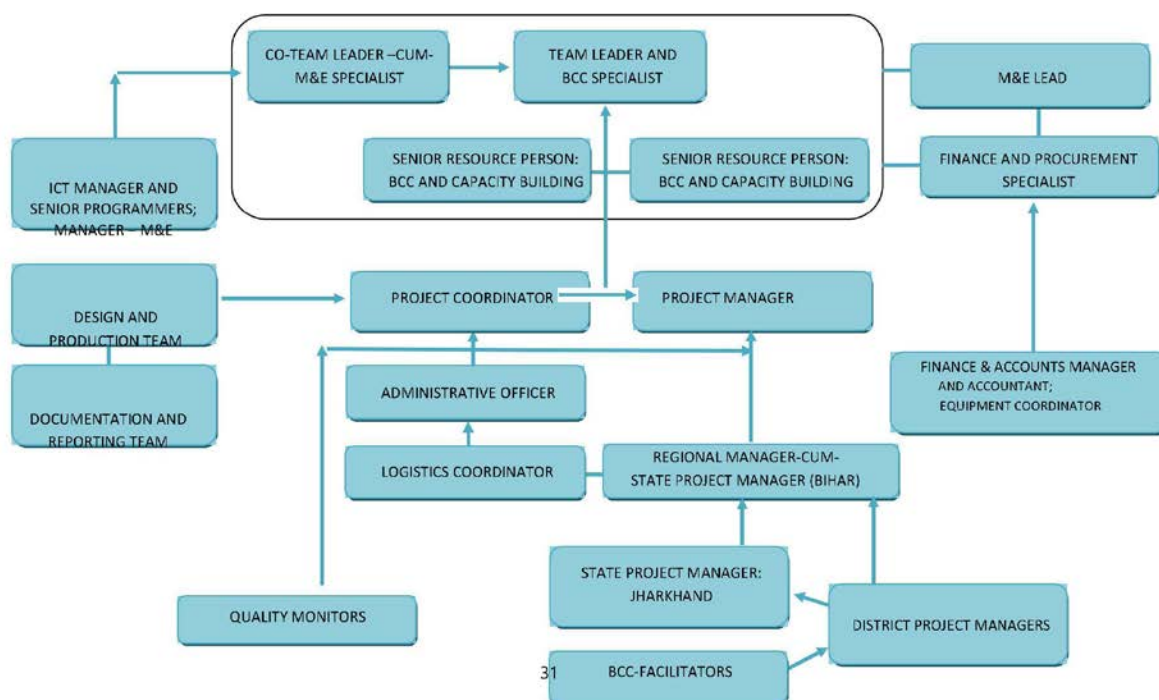

Supplement: 17-00087-Srinivasan-Supplement3.pdf [file 17-00087-Srinivasan-Supplement3.pdf]
