# Supplementary material for: Positive Influence of Behavior Change Communication on Knowledge, Attitudes, and Practices for Visceral Leishmaniasis/Kala-azar in India
Source: Glob Health Sci Pract. 2018 Mar 21;6(1):192–209. doi: 10.9745/GHSP-D-17-00087 (PMC5878072; doi:10.9745/GHSP-D-17-00087)
Supplement: 17-00087-Srinivasan-Supplement2.pdf [file 17-00087-Srinivasan-Supplement2.pdf]

Srinivasan R, Ahmad T, Raghavan V, Kaushik M, Pathak R. Positive influence of behavior change communication on knowledge, attitudes, and practices for visceral leishmaniasis/Kala-azar in India. *Glob Health Sci Pract*. 2018;6(1). https://doi.org/10.9745/GHSP-D-17-00087

SUPPLEMENT 2. Implementation Plan for BCC Intervention in Bihar, India

Level	Activities/ Materials	Audiences	Approximate No. of Activities	Who?	Coverage Targets	Rationale for Coverage Targets	Time Period
Village level	Group communication sessions	Community members, influencers, and frontline health workers	17,770 group communication sessions in 4,443 villages	BCC-Fs	At least 4 group communication sessions per village, conducted in every village and by follow-up activities	Group communication sessions should be conducted in at least 4 different wards within any particular village, where the most marginalized communities reside.	10 months
	VL film screening	School children (middle and secondary schools)	Screenings in 2,666 villages	BCC-Fs	Coverage of all villages which have middle/ secondary schools	At least 60% of all villages have a middle/secondary school, where this activity will be carried out	10 months
	FAQ booklet	Frontline health workers and community influencers	13,328 interactions in 4,443 villages	BCC-Fs	At least 3 interactions per village	At least 3 interactions per village, assuming that the FAQ booklet will be given at least to the frontline health workers and *Mukhiya*	10 months
	Flip-book	Community members (with involvement from frontline health workers)	44,425 IPC sessions in 4,443 villages	BCC-Fs	At least 10 IPC sessions per village	At least 8 IPC sessions per village, covering the wards where group communication sessions have not been conducted. The flip-book however, will be given to the frontline health workers.	10 months
	Interactive games and activities	Community members	Wherever possible	BCC-Fs	Wherever possible	Simple and participatory games/activities without using any BCC print or A/V material	10 months
	Posters on treatment	General public	44,425 posters	BCC-Fs	At least 10 per village	At least 1 poster in prominent locations within each ward	10 months
	Miking	General public	8,850 villages	Separate team	100% coverage	Miking during IRS rounds in all villages	10 months
	Wall stickers	General public	4,44,250 stickers	BCC-Fs	At least 100 per village	At least 10 stickers in prominent locations within each ward	10 months
Block level	Panels	General public	385	BCC-Fs	At least 1 per PHC (total 385 PHCs)	1 panel displayed in every PHC	10 months
	Interaction/ meetings with DVBDCOs, VBD consultants, and participating in forums	District officials	Flex (banner) in DVBDCO's office–132 (4 per year for 33 districts)	District project mgrs	Meetings to be ensured every week. At least 1 banner per district to be put up each quarter.	Flex of BCC activity updates being put up at the DVBDCO office will ensure BCC remains on their radar of activities and buy-in at the district level	11 months
	Display posters on vehicles plying in rural	General public	38,500	BCC-Fs	At least 100 per block	At least 100 display posters in vehicles. Not on buses as it will involve	10 months

Srinivasan R, Ahmad T, Raghavan V, Kaushik M, Pathak R. Positive influence of behavior change communication on knowledge, attitudes, and practices for visceral leishmaniasis/Kala-azar in India. *Glob Health Sci Pract*. 2018;6(1). https://doi.org/10.9745/GHSP-D-17-00087

Level	Activities/ Materials	Audiences	Approximate No. of Activities	Who?	Coverage Targets	Rationale for Coverage Targets	Time Period
	areas					buying space. Moreover, buses normally ply during night time only	
	Billboards	General public	33	BCC-Fs	Total 7 billboards (1 in each district)	At least 1 in each district, near the Sadar hospital or district manager's office. It is assumed that only govt. space will be used to install the billboards.	10 months
District and state level	Radio spot (only production)	General public	One 6- sec spot with 30-sec cutaway	NCIS	-	It is assumed media buying will come from the SHS/NHM budgets; based on which NCIS can share a media plan. NCIS will also help KalaCORE in advocating for getting media buying budget from SHS/NHM-Bihar.	10 months
	TV spot (only production)	General public	One 60-sec spot with 30-sec cutaway	NCIS	-		10 months
National level	Lesson-learning workshop at Patna	Development partners, program managers, and policy makers	1	NCIS	1 national lesson-learning workshop before the roll-out of BCC activities in Delhi	To share the achievements and lessons learnt during the preliminary phase and the way forward	10 months
	SMS alerts to frontline health workers, Gram *Pradhan/ Mukhiya*, select community members	Frontline health workers, Gram *Pradhan/Mukhiya*, select community members	1,250,000 SMS alerts	NCIS	SMS sent to ~25–30 per village throughout the project period. Assuming that all numbers are working and none are "do not disturb"	Serves as a reminder for key messages around VL symptoms, diagnosis, and treatment	10 months

Abbreviations: BCC, behavior change communication; BCC-F, BCC facilitator; FAQ, frequently asked questions; IPC, interpersonal communication; NCIS, New Concept Information Systems; NHM, National Health Mission; PHC, primary health center; SHS, State Health Society; VBD, vector-borne disease; VL, visceral leishmaniasis.
