# Supplementary material for: Positive Influence of Behavior Change Communication on Knowledge, Attitudes, and Practices for Visceral Leishmaniasis/Kala-azar in India
Source: Glob Health Sci Pract. 2018 Mar 21;6(1):192–209. doi: 10.9745/GHSP-D-17-00087 (PMC5878072; doi:10.9745/GHSP-D-17-00087)
Supplement: 17-00087-Srinivasan-Supplement1.pdf [file 17-00087-Srinivasan-Supplement1.pdf]

Srinivasan R, Ahmad T, Raghavan V, Kaushik M, Pathak R. Positive influence of behavior change communication on knowledge, attitudes, and practices for visceral leishmaniasis/Kala-azar in India. *Glob Health Sci Pract*. 2018;6(1).
https://doi.org/10.9745/GHSP-D-17-00087

SUPPLEMENT 1. Mapping of Communication Channels to BCC Activities, Materials, and Audiences

Channels	Activities	Materials	Description	Audiences
Mobile-based for knowledge and information dissemination	SMS alerts to frontline health workers, Gram *Pradhan/Mukhiya*, select community members		SMS messages around IRS and VL symptoms, diagnosis and treatment	Frontline health workers, Gram *Pradhan/Mukhiya*, select community members
IPC to reinforce positive messages	Group communication sessions		Holding interactive sessions with a group of 35-40 community members using VL film based on drama format	Community members, influencers, and frontline health workers
		Pen drive containing the VL film for group communication sessions	Film on VL prevention and treatment for conducting the group communication session<br><br>Minor changes will be made in the existing film, and this will be used for Bihar. A new separate film will be developed for Jharkhand.	Outreach team
		Diary for the BCC facilitator	A ready reckoner for the BCC facilitators to use as a guide/reference tool and document the field activities	BCC facilitators
	VL film screening		Screening the VL film for school children	School children (middle and secondary schools)
	Interactions	FAQ Booklet	For frontline health workers and community influencers to improve their knowledge on VL and to serve as a guide while interacting with community	Frontline health workers and community influencers
		Interactive games and activities	Simple and participatory games/activities, which can be carried out with the primary audience, without using any BCC print or A/V material	Community members, parents, and children
	IPC	Flip-book	For the frontline health workers during IPC sessions with community members	Frontline health workers
Outdoor to create a supportive environment		Posters	To be installed at prominent places the village for awareness generation	General public
		Wall stickers	To be put up on prominent walls in and around the health facilities and near main roads, with key messages pertaining to symptoms and treatment	General public
		Miking (only for Bihar)	To be carried out only in Bihar during IRS rounds	General public
		Drum beating (*Munadi*) (only for Jharkhand)	To be carried out only in Jharkhand during IRS rounds	General public
Outdoor/transit for awareness raising		Panels	To be displayed inside a PHC and/or Sadar hospital to create awareness among the community members visiting the facility	General public
		Display posters	To be displayed on rickshaws, tempos, and other vehicles plying in rural areas as transit media	General public
		Billboards	To be displayed at prominent places at district and block level	General public

Abbreviations: BCC, behavior change communication; FAQ, frequently asked questions; IPC, interpersonal communication; IRS, indoor residual spraying; PHC, primary health center; VL, visceral leishmaniasis.
