# Supplementary material for: Positive Influence of Behavior Change Communication on Knowledge, Attitudes, and Practices for Visceral Leishmaniasis/Kala-azar in India
Source: Glob Health Sci Pract. 2018 Mar 21;6(1):192–209. doi: 10.9745/GHSP-D-17-00087 (PMC5878072; doi:10.9745/GHSP-D-17-00087)
Supplement: 17-00087-Srinivasan-Supplement4.pdf [file 17-00087-Srinivasan-Supplement4.pdf]

Srinivasan R, Ahmad T, Raghavan V, Kaushik M, Pathak R. Positive influence of behavior change communication on knowledge, attitudes, and practices for visceral leishmaniasis/Kala-azar in India. *Glob Health Sci Pract*. 2018;6(1). https://doi.org/10.9745/GHSP-D-17-00087

SUPPLEMENT 4. Instructions for Field Implementation

1. ***Rapport building with key influencers:*** The BCC-Fs will meet the available village head (*Mukhiya*/ward member/*sarpanch*) and other workers such as ASHA, AWW; and / or any other key opinion leaders of the village to introduce themselves and orient them on the BCC activities which they plan to conduct. The support is also sought for:
    a. Their opinion on the wards (*tola* and hamlets) where KA cases have been reported and therefore need to be given priority
    b. Identification of the venue for GC sessions
    c. Informing the villagers (especially women; and other vulnerable groups) about the date, time and venue; and identifying a set of motivated adult men and women to participate actively in the GC sessions
    d. Identifying potential Kala-azar cases within the community, referring them to the nearest PHC through ASHAs, and follow up

2. ***Group communication sessions using the KA film:*** Trained BCC-Fs (in pairs) will conduct GC sessions using the film in identified KA-affected villages – with the key objective of providing information on Kala-azar to the community and bringing about community-level action and behaviour change towards ensuring early diagnosis, treatment through public health delivery channels, and prevention in the entire village. A GC session will take around 1.5-2 hours.

    Villages for holding GC sessions will be selected based on certain norms, such as Kala-azar endemicity, population of the village, presence of an active ASHA and presence of marginalised communities such as *Musahars, Bin, Nonia* and other marginalised groups. Depending on the village size and presence of marginalised communities, it is estimated that at least four GC sessions will be conducted in each village. A conscious attempt will be made to conduct GC sessions in *Musahar tola* and ensure ASHA's presence in every GC session.

    The BCC-Fs will conduct the GC session at a specific ward (which will be communicated in advance to the villagers). Each GC session will have a core group of identified 25-30 motivated community members within a *tola* – and who are specially mobilised for the GC session. This will comprise a mix of men and women, motivated / educated adolescent girls and boys and key influencers like the *Mukhiya*, ward member, ASHA and the village functionaries, if any.

    The KA film contains break points with a set of questions for discussions with the community. Answers for each of these questions will be brought out through these discussions, with clarifications sought wherever required. All key messages will be reiterated at the end and the community will be encouraged to make an action plan for its village to become Kala-azar free.

3. ***IPC sessions with the community using the flip book; interactive games and activities:*** The BCC-Fs will mobilise a small group at the community level (max 7-8 people) and carry out an IPC session with them. The duration of the session will vary from 15 mins focusing on a specific topic (like symptoms of Kala-azar, early diagnosis or treatment using AmBisome) or about 45-60 mins to discuss the entire flip book. The facilitator will also discuss the questions and reiterate the key messages after the IPC session. Efforts will be made to conduct it in the presence of the ASHA or AWW.

After the IPC session, the facilitator will share the flip book with the ASHA and any other village-level functionary in each village. The BCC-F will explain what the flip book is about, take them through the contents and orient them on how to use the material with the community for information dissemination. It will be reiterated that the flip book is a tool to be used for IPC sessions.

In addition to using flip books, efforts will be made to develop interactive games / activities to be used for effective IPC.

4. ***Interaction with the ASHA and other community-level influencers using the FAQ booklet:*** Besides being oriented on the use of flip books, ASHAs will be given FAQ booklets as a reference tool. In addition to ASHAs, key influencers (like the *Mukhiya*, ward member, etc.) in the community with a certain level of education will be given the FAQ booklet to educate themselves to inform others.

   The BCC-Fs will explain to them what the FAQ booklet is about, take them through the contents and orient them on how to use the material with the community for information dissemination. It will be reiterated that the FAQ booklet is to be used as a reference tool and should be shared with other educated community members, too.

5. ***Spreading information using posters and stickers at the community and block level:*** The BCC tools will include a poster on treatment and stickers on symptoms for the community. The BCC-Fs will share the poster with the ASHA and any other village-level representative like the *Mukhiya*. She/he will explain to them what the poster is about and its contents and assign them the responsibility for taking care of it. With help from the BCC-F, the poster(s) is placed in a proper area like *Mukhiya's* house/wall, community gathering area, school, AWC, panchayat bhavan, etc. Through the poster, even the community members will get to know about single-dose AmBisome treatment at the Sadar hospital so that they can spread the word.

   Stickers can be put up at many more prominent places within the village and one or two at the entrance of the village. These can also be used in transit media – in tempos, autos, etc. which ply in the rural areas. The BCC-Fs can receive permission from the *Mukhiya* to put up the stickers at various locations within the village.

   These two products can be placed at various prominent and important locations within the village (and hamlets) such as AWC, panchayat bhawan, entry and / or exit points of the village, walls of houses, etc.

6. ***KA film screening in schools:*** In villages which have upper primary / secondary / higher secondary schools, the BCC-Fs will screen the Kala-azar film (using the TV set) for children in the school. The BCC-Fs ensure that the school principal or headmaster is present during the screening and invite him / her to encourage the children to spread key messages for Kala-azar elimination.

7. ***SMS Alerts:*** The main objective behind sending the SMS alert is to motivate / remind the community about Kala-azar – causes, symptoms, diagnosis, treatment and prevention (IRS). The database will be collected through the BCC-Fs when they go to each village for conducting BCC activities. These will be compiled to generate the database sending SMS alerts – which will include the village *Mukhiya*, ASHA, AWW and other village level functionaries.

Srinivasan R, Ahmad T, Raghavan V, Kaushik M, Pathak R. Positive influence of behavior change communication on knowledge, attitudes, and practices for visceral leishmaniasis/Kala-azar in India. *Glob Health Sci Pract*. 2018;6(1). https://doi.org/10.9745/GHSP-D-17-00087

The SMS will only be sent from 9:00 AM to 9:00 PM as per TRAI guidelines and will be made 1-2 days before the IRS, specifically to the village-level influencers and functionaries. As follow-up, a call will be made to some of them randomly (approx. 5% of the total numbers) to check if they have received any SMS on Kala-azar.

8. ***Follow-up of BCC activities:*** The BCC-Fs will visit the village to follow up on status of community-level action points which came out of the GC sessions. In addition they will also follow up on the present database of potential KA cases with the ASHA of the respective village, as well as the respective MoIC, so that follow-up can be carried out for detection, diagnosis and treatment at the Sadar hospital (if found KA-positive).

Photographic evidences as well as reporting formats will capture the interactions and names and mobile numbers of the frontline functionaries, village members who receive and/or participate in BCC sessions as proof of evidence. These formats will be finalised in consultation with the KalaCORE team.
